# How to grow a self: development of self-representation in the Bayesian brain

**DOI:** 10.3389/fnhum.2024.1441931

**Published:** 2024-11-18

**Authors:** Mateusz Woźniak

**Affiliations:** ^1^Social Cognition in Human-Robot Interaction Group, Italian Institute of Technology, Genoa, Italy; ^2^Social Mind Center, Department of Cognitive Science, Central European University, Vienna, Austria; ^3^Cognition and Philosophy Lab, Department of Philosophy, Monash University, Melbourne, VIC, Australia; ^4^Institute of Psychology, Jagiellonian University, Cracow, Poland

**Keywords:** self, self-representation, Bayesian brain, predictive coding, cognitive development, self-recognition, bodily self, abstract self

## Abstract

The last two decades saw multiple attempts to explain how the self is represented in the brain within the framework of the Bayesian brain. However, these attempts largely focused on describing a developed, adult self-representation. The current paper argues that looking at the developmental trajectory is crucial for understanding the structure of self-representation. It argues that emergence of self-representations should be understood as an instance of the process of acquisition of new internal models of hidden causes of sensory input. The paper proposes how such models emerge and develop over the course of life by looking at different stages of development of bodily and extra-bodily self-representations. It argues that the self arises gradually in a series of discrete steps: from first-person multisensory representations of one’s body to third-person multisensory body representation, and from basic forms of the extended and social selves to progressively more complex forms of abstract self-representation. It discusses how each of them might emerge based on domain-general learning mechanisms, while also taking into account the potential role of innate representations. Finally, it discusses how predictions of the proposed model might be experimentally tested.

## 1 Introduction

What is the self? William James (1890) revolutionized the discussion about this question and set foundations for scientific study of the self by introducing the distinction between self-as-subject (“I”) and the self-as-object (“Me”). The importance of this idea lies in the fact that while the problem of “I” is a metaphysical problem (James, 1890; Woźniak, 2018), the “Me” can be investigated as either a type of experience, or as an underlying structure of mental representations (self-models).^1^ Arguably, this insight shifted the problem of the self from being a purely philosophical issue into a scientific research question which can be approached by empirical science through investigation of neural and cognitive underpinnings of our self-representations. Afterwards, research on the self entered the era during which it has been investigated as a psychodynamical mechanism (Freud, 1933), symbolic knowledge (Baumeister, 1999; Markus, 1977; Mead, 1934; Tajfel, 1982), and a connectionist network (Kihlstrom et al., 2003; Smith et al., 1999).

During the last decade a number of proposals attempted to explain the self as a neurocognitive structure within the predictive (Bayesian) brain. These theories typically (but not always) refer to more specific frameworks of predictive coding and the free energy principle (Friston, 2005, 2010) and target several important aspects of the self, such as body representation (Apps and Tsakiris, 2014; De Vignemont, 2010; Hohwy, 2007, 2013; Limanowski, 2022; Limanowski and Blankenburg, 2013; Salomon, 2017; Seth, 2013), the role of interoception (Allen and Tsakiris, 2018; Fotopoulou and Tsakiris, 2017; Seth, 2013; Seth and Tsakiris, 2018), abstract and social aspects of self-knowledge (Allen and Tsakiris, 2018; Bolis and Schilbach, 2020; Fotopoulou, 2012; Fotopoulou and Tsakiris, 2017; Friston and Frith, 2015; Hohwy and Michael, 2017; Moutoussis et al., 2014), as well as the self as present in conscious experience (Ciaunica, Constant, et al., 2021; Ciaunica, Safron, et al., 2021; Letheby and Gerrans, 2017; Seth et al., 2011; Woźniak, 2018).

Most Bayesian notions of the self focus on the adult self. They describe the structure and dynamics of something that has been already formed throughout development. As such, they tend to omit an important aspect of the problem – the question of how the self emerges and changes during life. However, in the recent years several papers have introduced the developmental perspective into this field. Frederique de Vignemont (2010) tackled developmental issues when discussing emergence of body representations and Anna Ciaunica and colleagues recently stressed the importance of looking at the development when explaining the origins of self-consciousness (Ciaunica et al., 2021a; Ciaunica and Crucianelli, 2019; Ciaunica et al., 2021b). Katarina Fotopoulou and Manos Tsakiris used the developmental perspective to illuminate specific aspects of development of the self such as self-recognition (Drysdale and Tsakiris, 2021) and the role of “mentalization” in constituting one’s self (Fotopoulou and Tsakiris, 2017). Finally, Riva (2018) has recently proposed a comprehensive developmental model of bodily self-representation in which he outlined a series of stages during which an adult body-representation comes into being.

This paper aims to introduce a different, partially complementary and partially competing, perspective into this field and propose a mechanistic model of development of self-representation within the framework of the Bayesian brain. The first goal of this paper is to discuss a developmental mechanism of acquisition of new internal models. The second goal is to use it to propose a potential trajectory of development of an adult self-representation. The resulting proposal bears many similarities to Riva’s (2018) model and the final section will discuss differences and similarities between these two (as well as other) theories.

The current proposal focuses on self-representation (how our mind/brain represents ourselves) and not on self-consciousness (how we consciously experience ourselves). It takes a representationalist approach (for discussion of what is a representation in cognitive science and predictive coding theories see: Baker et al., 2022; Egan, 2012; Ramsey, 2016; Williams, 2018) and assumes that self-representations underpin conscious self-experience, but are not synonymous with it, as many self-representations are unconscious. On the other hand, this approach also assumes that any self-related conscious experience must be underpinned by some form of self-representation, meaning that research on self-consciousness remains directly relevant to the discussion of self-representation. Finally, this paper limits itself to presentation of the theory at the computational level (Marr, 1982), although it also refers to recent developments which can illuminate it on algorithmic and implementation levels.

I will describe the model mostly through the lens of the predictive coding framework (Friston, 2005; Rao and Ballard, 1999; Spratling, 2016, 2017, for discussion of neural implementation see: Bastos et al., 2012; Keller and Mrsic-Flogel, 2018; Shipp, 2016), although it is equally compatible with other Bayesian and predictive models of cognition (Fiser et al., 2010; Griffiths et al., 2010; Knill and Pouget, 2004; Orbán et al., 2016; Tenenbaum et al., 2011, also: Heeger, 2017), as well as with connectionist models of cognition (DiCarlo et al., 2012; Kriegeskorte, 2015; Kumaran et al., 2016; McClelland et al., 2010; McClelland and Rogers, 2003; Yamins and DiCarlo, 2016).

The paper is divided into two parts. The first part will clarify the theoretical background by (1) specifying what I here will understand as self-representation, (2) describing basic postulates of the Bayesian model of the mind, and (3) explaining how to understand cognitive development within the Bayesian framework. The second part of the paper will then propose a model of cognitive development of self-representation.

## 2 Self-representation, Bayesian brain, and acquisition of new models

### 2.1 The self as a representational structure

The goal of this paper is to propose a theory of development of the self understood as self-representation Bayesian approaches often assume a representational view of the mind (Hohwy, 2013, but see: Clark, 2016; Williams, 2018). In line with this assumption, when I speak about the self, I refer to a representational structure, which is encoded in the brain. Aspects of this structure can become conscious, but in principle mental representations can be analyzed independently of whether they are conscious or not (Chalmers, 2004; Crane, 2003).

**Figure 1:**
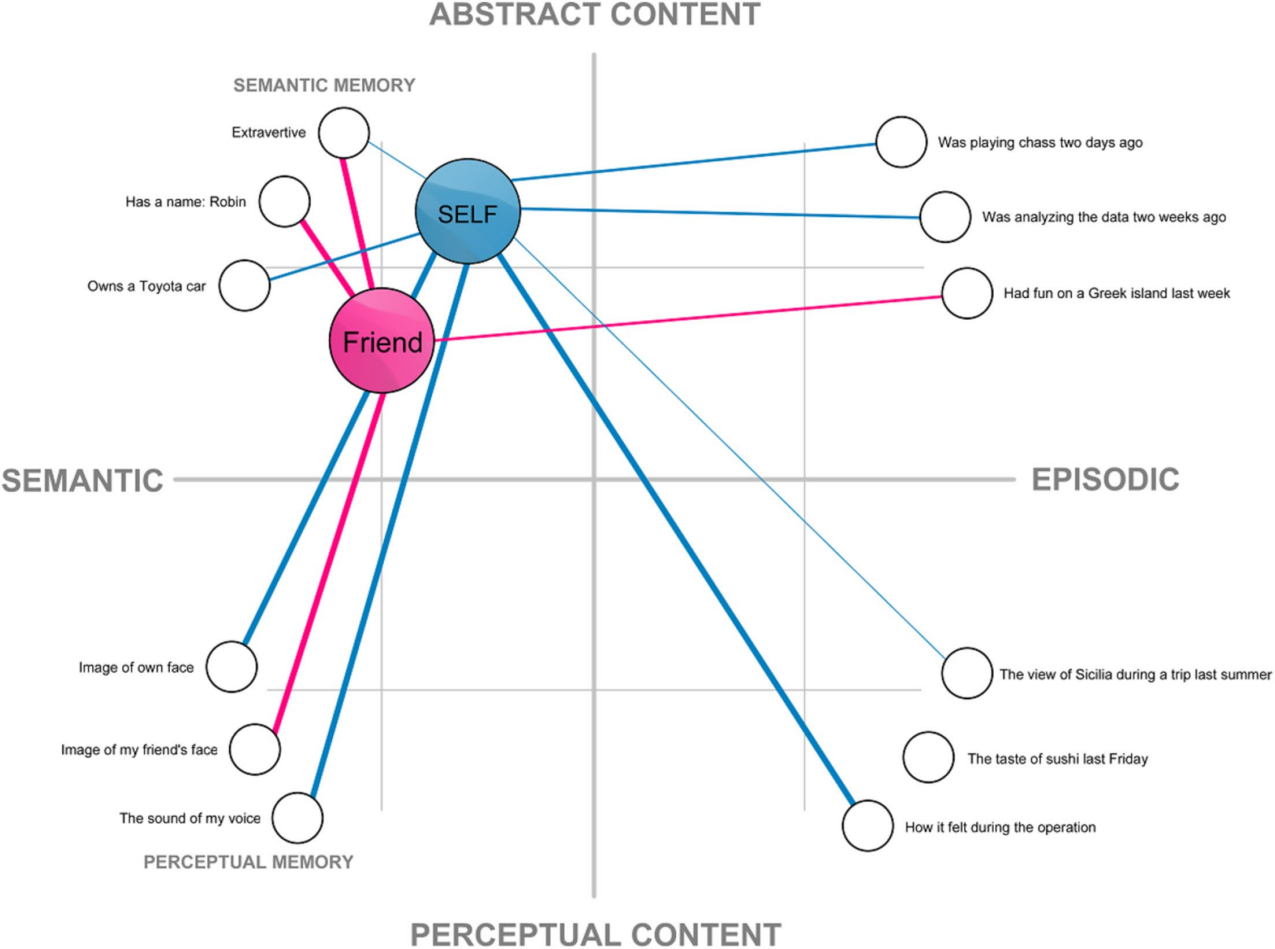
An example of a localist connectionist model of self-representation based on the associative network model of memory (cf. Kihlstrom et al., 2003; Smith et al., 1999). Mental representations are presented as nodes on a two dimensional plane spanning perceptual-to-abstract content and semantic-to-episodic content. The internal model of the self and a friend are visualized as elements of the semantic memory. These nodes are connected with other representations. Thicker lines denote strong connections between the nodes, while thinner lines represent weaker ones.

This understanding of the self has been implicitly assumed in much of traditional research in cognitive science and psychology, especially within the connectionist framework, in which self-representation is understood as a structure in memory (e.g., Kihlstrom et al., 2003; Smith et al., 1999, but also: Conway, 2005; Hart et al., 1997; Haslam et al., 2011; Martinelli et al., 2013). The connectionist approach can provide a useful way of conceptualizing self-representation as a network composed of: (a) nodes representing memory content, which are linked to (b) a node representing the internal model of the self (Figure 1). Under such conceptualization, what makes my mental representation of my face a part of self-representation is the fact that it is linked with my self-model, while my representation of another face (my friend’s) is not. A connectionist network provides a convenient way to visualize all aspects of the self-representation on a two-dimensional plane. However, as will be argued later, the structure of the self is more complex.

### 2.2 The self as an internal model

In Bayesian and predictive models the brain is seen as an inference machine which continuously attempts to explain the world by combining sensory input (data in Bayesian terms, or prediction error in predictive coding framework) with prior knowledge (priors or predictions, respectively) in order to yield a posterior representation (or more properly: posterior distribution) which is responsible for the eventual percept. It can be illustrated with an example. Let us say that I invited two of my friends, Anna and Julia, for dinner at 7 pm. Exactly at 7 pm I hear a door bell. I know that Anna always comes on time, while Julia is well-known for being late. Given this prior knowledge, I am certain that it is Anna waiting at the door. I open the door and it takes me a while to realize that I’m standing in front of Julia instead. Under Bayesian interpretation, I had a very strong prior belief that I will encounter Anna. However, the sensory input strongly suggested otherwise because I saw a person looking like Julia at the door. Sensory data eventually overcame my prior belief and led to the formation of a posterior belief that it was Julia that came first, and not Anna.

Conducting this type of (Bayesian) inference requires having a hypothesis space. In its simplest form it can be just a collection of individual hypotheses (a categorical distribution). In the example above there were only two hypotheses: that a person at the door is Anna or Julia, but one can entertain more hypotheses, e.g., that it is a postman or a neighbor^2^. Hypothesis spaces can also have a continuous form, e.g., when I estimate the height of a person (what can be parametrized as a normal distribution) or somebody’s number of Facebook friends (typically following a power-law distribution). Regardless of the form of the hypothesis space, in order to calculate the posterior distribution using Bayesian inference, I need to have models (representations) of possible causes. For example, if I open the door and see a wallaby there, I will not be able to recognize it as what it is, until I possess an internal model of a wallaby. If I do not have such model then I may try to accommodate what I see into the closest of my existing models - in this case, for example, my internal model of a kangaroo.

**Figure 2:**
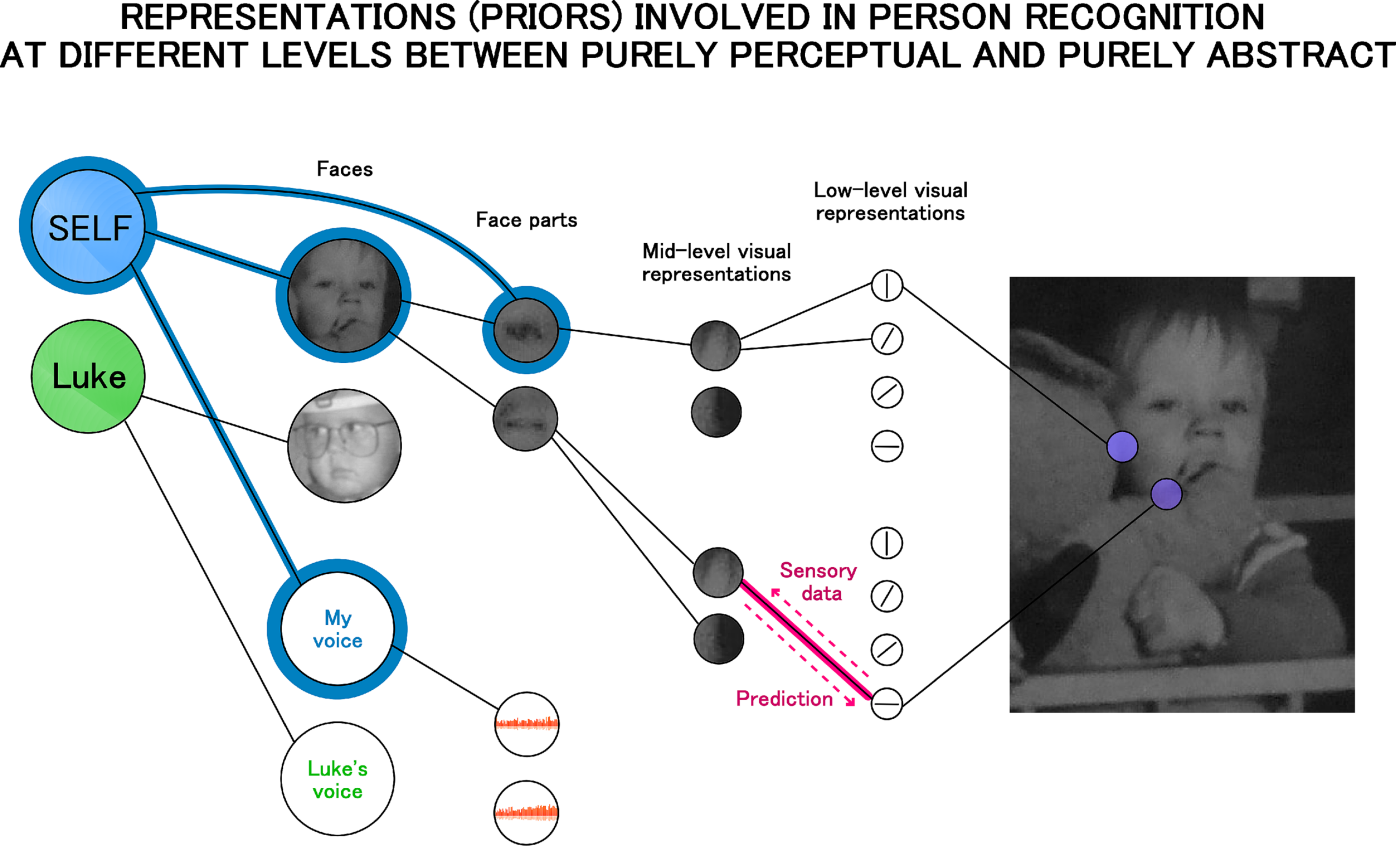
Self-representation as a hierarchical structure of internal models with the self-model as a high level abstract prior (on the left). Each link in the hierarchical structure reflects the interplay of top-down and bottom-up signals as illustrated by the link in pink. In Bayesian terms the top-down signal reflects prediction or strength of the prior, and the bottom-up direction reflects prediction error or the incoming data. The self-model (the blue node) is connected to the representation of a person’s voice and face, but also to the representation of one’s eye illustrating that representations are free to be connected across levels of the hierarchy.

Under predictive coding and other Bayesian models the brain is understood as a network of internal (generative) models representing hidden causes of sensory input. This network is organized in a hierarchical manner. The most abstract causes are at the top (high-level, including social, semantic categories which are represented in the anterior temporal lobes, orbitofrontal cortex, and ventral medial prefrontal cortex: Bowman and Zeithamova, 2018; Chen et al., 2017; Clarke and Tyler, 2015; Ralph et al., 2017) and the most basic perceptual ones at the bottom (e.g., models representing line orientations on a patch of the retinal input are represented in primary visual cortex: Hubel and Wiesel, 1962, 1968). According to some predictive coding theories the whole brain is organized in this way (Friston, 2010), while others attribute this architecture only to the cortex (e.g., Friston, 2005; Spratling, 2010). Nevertheless, both views imply that self-representation must be underpinned by this architecture as well, and the theory proposed here shares this assumption. Figure 2 illustrates it graphically. It provides an elaboration of the connectionist conceptualization of the self, but also includes visualization of the hierarchical structure of internal models (representations).

### 2.3 Learning new internal models

Across lifespan people not only use representations with which they were born (for a discussion see: Bottari et al., 2015; Carey, 2009; Heyes, 2018; Reid et al., 2017), but also acquire new ones. In the Bayesian framework this raises the problem of how we come to acquire new internal models (representations). If I see a person in front of me I will assume that I am facing a living human being. However, if I possess an internal model of a TV then I may be capable of forming an alternative hypothesis – that the human figure in front of me is not a real person, but just a video recording played on a TV screen. Acquiring new models allows us to expand the hypothesis space and, as a consequence, allows more hidden causes to be taken into account when trying to make sense of a given situation.

**Figure 3:**
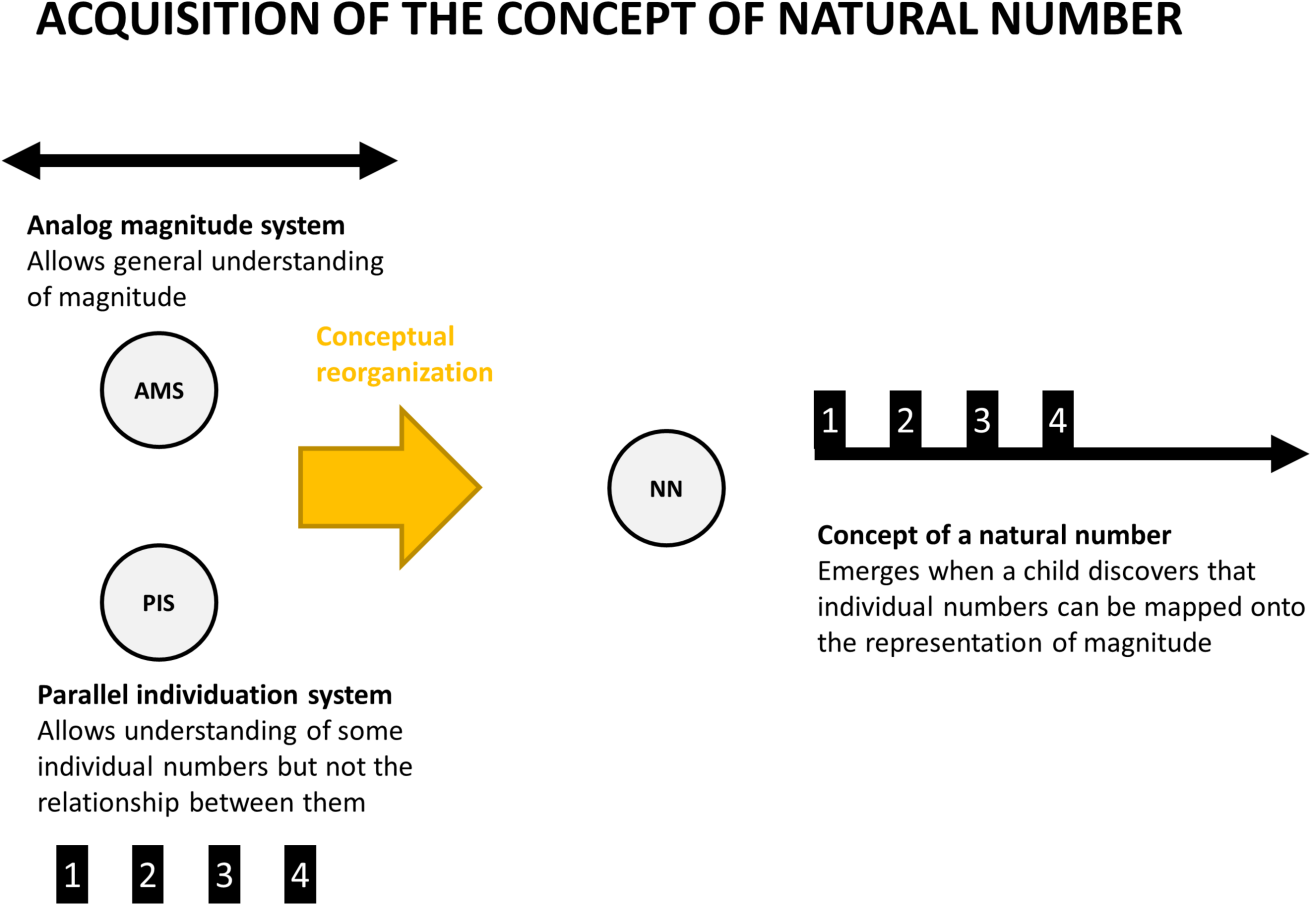
A graphical illustration of the process of acquisition of the concept of a natural number (NN). According to Carey (2009) children are born with an innate concept of magnitude (through “Analog Magnitude System,” or AMS) which allows them to differentiate between, e.g., long and short objects. Children are also born with a “Parallel Individuation System” through which during the third year of life children begin to understand the concept of individual numbers: one, two, three, sometimes four. The crucial point of Carey’s proposal is that at certain point children discover that individual numbers can be mapped onto the underlying representation of magnitude (Carey proposes that it happens through the mechanism of Quinian bootstrapping) and hence acquire the concept of a natural number.

Susan Carey (2009) has proposed an influential theory of development of understanding of concepts. While she focused on concepts, her proposal can be applied to development of new internal models in general. In her view conceptual development is a discontinuous process consisting of episodes of qualitative change, during which the existing concepts (representations) are recombined into new, more powerful ones.^3^ She illustrates it with an example of acquiring the concept of a natural number by human infants. Until 2 years old, most children do not seem to understand the concept of a number at all. Then, usually between 24 and 30 months of age, they begin to understand the concept of “one.” Approximately 6 to 9 months later they begin to grasp the concept of two, but still fail with larger numbers. Later on, they become “three-knowers,” and sometimes also “four-knowers,” but around that time most of them undergo a qualitative change in their conceptual representation of numerosity, which leads them to understanding of the concept of a natural number (what happens typically when they are between 3 and 6 years old). Carey explains this transition by proposing that children recombine their primitive representations (structures which are part of the innate core knowledge, in this case the “parallel individuation system” and the “analog magnitude system”) through a process called “Quinian bootstrapping” (Figure 3). It is not necessary to commit to the existence of an innate stock of representations, nor to the claim that conceptual change can happen only through Quinian bootstrapping, to grasp the main idea behind her proposal: we acquire new concepts by recombining pre-existing representations, what leads to the emergence of new mental representations which are incommensurable with the old ones. In case of the natural numbers: we begin with understanding of the concept of “one,” then “two” and “three,” and then at some point we discover the underlying rule by mapping them onto our representation of magnitude. The discovery of that rule leads to mental reorganization which translates to discovery of the concept of a natural number. At later stages children and adults can recombine this concept of a natural number with other concepts to acquire even more abstract concepts like integer or rational number, and even to revisit the concept of a natural number from a different perspective, e.g., when deliberating whether zero is a natural number or not.

If we apply Carey’s idea to the Bayesian brain framework, then development of new internal models can be understood as the process of restructuring of a hypothesis space leading to the emergence of new internal models (cf. Perfors et al., 2011; Tenenbaum et al., 2011; Ullman and Tenenbaum, 2020) This idea has been recently developed in computational models attempting to model the process of discovery of new categories (Love et al., 2004; Navarro and Kemp, 2017; Pothos and Chater, 2002; Smith et al., 2019). One prominent example of such process has been illustrated in models of learning of the structural form of data (Kemp and Tenenbaum, 2008; Lake et al., 2018), but for our purposes a more relevant example has been provided by Hohwy and Michael (2017), who wrote about a similar process in the context of self-recognition. Hohwy and Michael described a situation of a human organism, which detects brief periods of darkness every few seconds. One possible cause of this situation is that the light goes off and on. But there is an alternative explanation once one realizes that brief periods of darkness are perfectly correlated with motor commands sent to one’s eye lids. As such, one may come up with a second explanation, that it’s not the world, but oneself that is switching the light on and off by opening and closing the eyes! However, in order to take this hypothesis into consideration one needs to possess an internal model of oneself as a hidden cause of sensory input, i.e., a model of the self. In other words, in order for me to be able to realize that a hidden cause of my sensory input is “me,” I need to possess at least a rudimentary model that such a hidden cause as “me” exists. The remaining part of the paper will introduce a proposal of how such a model emerges and how it develops across lifespan (Figure 3).

## 3 Development of the self

Previous sections introduced a picture of the brain as a Bayesian inference machine and described the place of the self in its architecture. It can be summarized as follows:

1. The brain can be understood as a Bayesian hierarchical inference machine, which attempts to model the environment by inferring hidden causes of sensory input. The self-model is just one model of such hidden causes.
2. Self-representation can be understood as a network composed of (a) a central node representing the self-model (which is a representation of a hidden cause, i.e., oneself), linked with (b) multiple other representations (other hierarchical representations of hidden causes, e.g., own arm, own hand, own finger, one’s nationality, one’s autobiographic memories, etc.).
3. Cognitive development is a process of acquiring new representations, i.e., new internal models, through a process of recombination of the existing representations. Here, I do not specify the mechanism which allows the brain to do it, but I will suggest some of them when discussing individual developmental steps.

**Figure 4:**
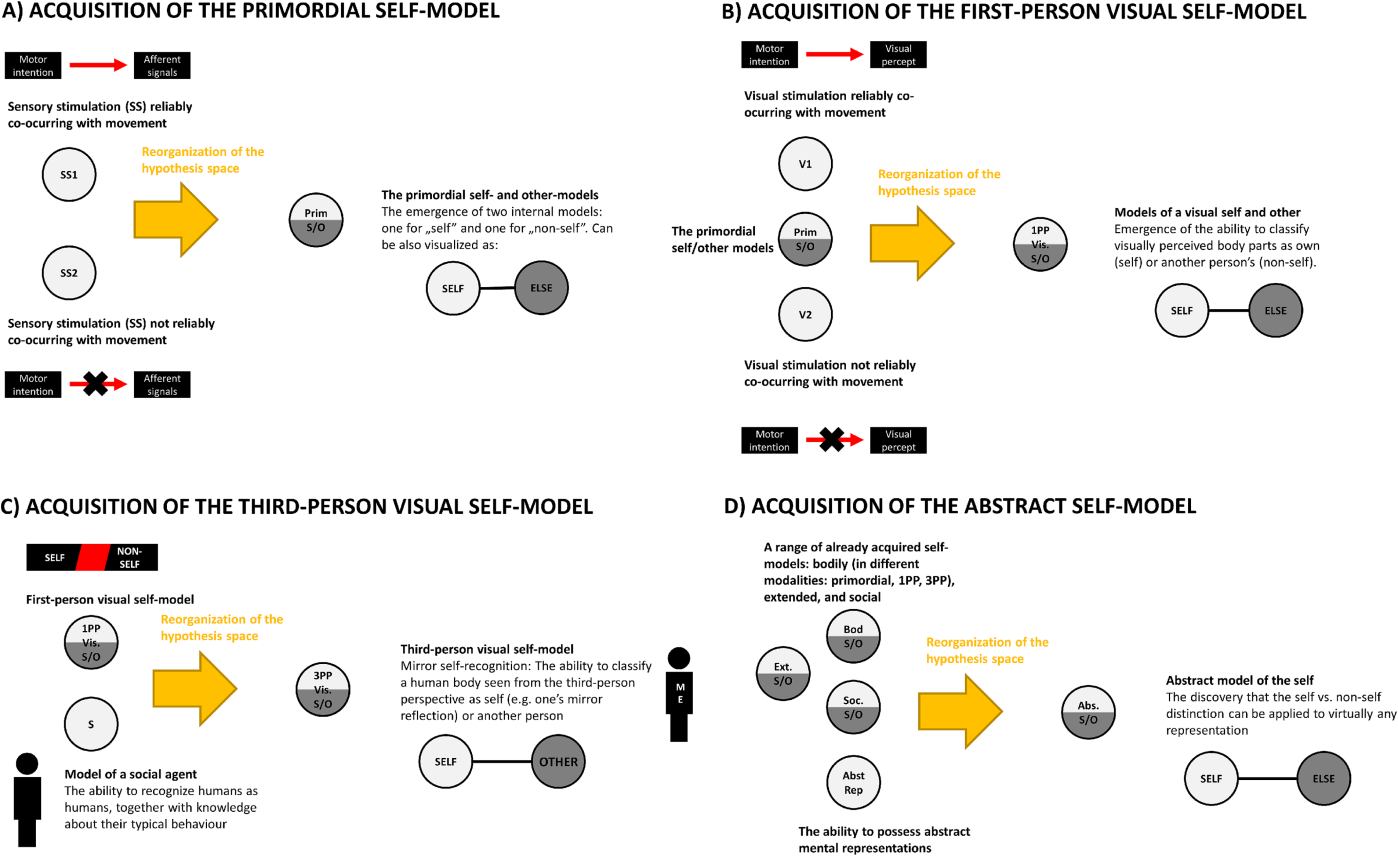
Graphical illustration of acquisition of four types of self-models. In each case the existing representations/models (shown on the left in each section) are recombined (indicated by the yellow arrows) into novel self-models (shown on the right). **(A)** The primordial self-model emerges once the brain discovers that it can classify sensory signals into the ones which are reliably predicted by one’s motor behaviour and those which are not. **(B)** The same classification rule can be applied to visual perception leading to acquisition of first-person representation of one’s body. **(C)** A toddler that possesses the concept of another person and understands that some visual percepts represent their body can discover that in special cases (e.g., encounter with a mirror) they can come together, and that it is possible to see oneself from the third-person perspective. **(D)** An abstract self-model emerges once a child discovers that self/non-self distinction can be applied to abstract representations.

Based on these assumptions the rest of the paper will outline a proposal of the developmental trajectory of the self, which is understood as a representational structure composed of multiple internal models. I will argue that the adult human self-representation emerges as a consequence of a series of discrete developmental steps, beginning with the emergence of a primordial form of the self-model and ending with a mature form of an abstract self-model. Figure 4 provides a brief summary of the main postulated developmental steps.

### 3.1 The primordial self: sensorimotor and interoceptive body representations

It is typically assumed that a person is born after leaving the womb. However, birth follows a 9-month long period of intensive prenatal development during which the neural system is being formed and organized (Anderson and Thomason, 2013; Kostović and Judaš, 2010; Moore and Linthicum Jr, 2007). Moreover, even before birth foetuses show a wide range of spontaneous behaviors, react to stimulation in a wide range of sensory modalities, and even exhibit behavioral displays of learning (Anderson and Thomason, 2013; Hepper, 2015). Given all of these, a full account of cognitive development, including development of the self, must also take into account processes which take place during the foetal period of life (Ciaunica et al., 2021a; Ciaunica et al., 2021b).

**Figure 5:**
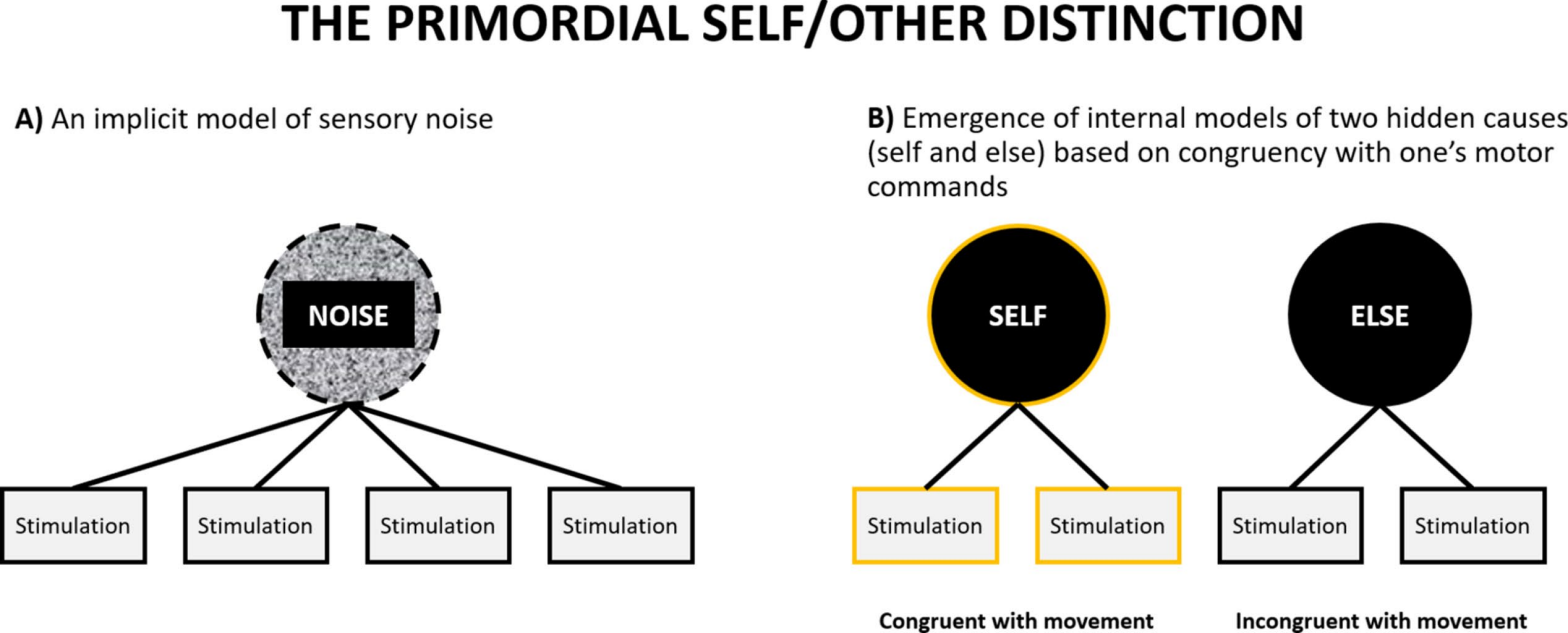
A schematic illustration of emergence of the primordial self-model. **(A)** At the outset an organism has only an implicit model of sensory noise. **(B)** The discovery that some sensory stimulation reliably co-occurs with one’s motor commands leads to the emergence of the primordial models of self and else (see also Figure 4A).

Can a primordial self-model emerge during prenatal development? Previous theories postulating the importance of sensorimotor integration for the emergence of the self have suggested how such a process might take place (although within a non-representationalist framework: Christoff et al., 2011; Legrand and Ruby, 2009). Let us assume an idealized scenario in which a foetus has already developed some basic sensory (tactile, proprioceptive, nociceptive, visceral) systems which allow their brain to detect sensory stimulation (without claiming that it happens consciously). If we idealize and assume that at that point their brain does not yet have any models of external causes of this stimulation, we may compare this situation to experiencing a multisensory perceptual noise – something similar to hearing a uniform white noise signal, but across many sensory modalities. It is a situation in which one cannot make sense of anything in the environment, because one does not have any models of what can be out there. The only thing that one has is an implicit model to account for everything that happens in the environment: the model of sensory noise (Figure 5A). Second, let us also assume that the foetus has a rudimentary motor system, which allows them to initiate random movements (indeed foetuses exhibit a rich repertoire of motor behaviors: Arduini et al., 2013; DiPietro et al., 1998; James et al., 2013; Robertson, 1990). Each such movement will be accompanied by some pattern of sensory consequences. For example, performing a random hand movement will lead to a specific tactile and proprioceptive pattern of sensations, and performing a leg movement will lead to a different pattern. In both cases these patterns will be structured in a different way than any pattern of stimulation elicited by external sources, such as movements of their mother’s body. However, the pattern of sensory stimulation accompanying one’s movements (that will come as a consequence of initiating a motor command) will covary with one’s movement with much greater regularity than the pattern of stimulation caused by the mother. This constitutes a reinterpretation of the notion of self-specifying processes discussed by Christoff et al. (2011). Similar models have been also proposed in developmental robotics (Hoffmann, 2022; Schillaci et al., 2016).

At this point the task of the brain is to pick up on this regularity and perform something akin to cluster analysis. It needs to utilize the statistical regularity between motor commands (or more specifically: patterns of neural activation in the motor system, even if they are generated randomly) and their sensory consequences (patterns of neural activation in the sensory systems) and discover that it can probabilistically classify the sensory signal into two categories: something that can, and something that cannot be predicted by motor activity. This might result in the emergence of two categories at the top level of the sensory hierarchy, and any sensation can thereafter be classified as one of these two: something caused by me or something caused by something else. Although the foetus will likely not be aware of the meaning of this classification, it will mark the emergence of the fundamental distinction between the self and the environment, and representations of the corresponding categories can be regarded as primordial models of the self and environment.

Within the Bayesian framework this situation can be rephrased as a simple instance of formation of models of two hidden causes,^4^ based on statistical regularities detected in the sensory input. As such, it can be regarded as one of the simplest instances of new model acquisition and is in agreement with Bayesian computational models of the brain (Lake et al., 2018; Perfors et al., 2011; Tenenbaum et al., 2011; Ullman and Tenenbaum, 2020). However, is the prenatal brain capable of implementing such an operation? It appears that yes. Twins *in utero* show developmental differences between touching behavior toward themselves, the other twin, and the uterine wall (Castiello et al., 2010). These results suggest that foetuses can not only discriminate between self and the uterine environment, but also between self and another foetus. There is also evidence that foetuses can learn to discriminate between the voice of mother and other people (DeCasper and Fifer, 1980; DeCasper et al., 1994; Kisilevsky et al., 2003), what presents a much more difficult problem requiring much finer discrimination than a simple ability to distinguish between self-caused and externally caused sensations. Second, computational models of human brain can easily learn much more sophisticated multidimensional discriminations (e.g., Lake et al., 2018; Smith et al., 2019). For example, based on earlier computational models it has been argued that emergence of receptive fields in the visual cortex can be understood as an instance of unsupervised learning over visual input, which leads to performance of something akin to principal components analysis (Olshausen and Field, 1996; Simoncelli and Olshausen, 2001). In this context, forming two models reflecting self and other seems to be a trivial task, even for a brain at a very early stage of development.

It is difficult to overestimate the usefulness for survival of being able to perform this distinction. Therefore, it is highly plausible that most living organisms which possess nervous system are evolutionarily pre-equipped with it. Regardless of whether it is acquired through learning or as a part of innate core cognition, it appears that a newborn child enters the world with a basic ability to differentiate between self and everything else, an ability which, under the Bayesian account, is underpinned by existence of specific models of self and else.

This view of the primordial self builds on and is in agreement with theories proposed previously by other authors which emphasize that the self emerges on the basis of sensorimotor signals (Blanke, 2012; Blanke and Metzinger, 2009; de Klerk et al., 2021; Gallagher, 2000; Gallagher, 2006; Salomon, 2017), and especially the ones which treat the sensorimotor loop as a self-specifying process (Christoff et al., 2011; Legrand and Ruby, 2009). Just like these previous proposals it postulates that sensorimotor congruency is fundamental for the emergence of the most basic form of self-representation. It is also in partial agreement with theories that draw attention to the role of interoception for the self (Allen and Tsakiris, 2018; Babo-Rebelo and Tallon-Baudry, 2018; Damasio, 1999; Park and Tallon-Baudry, 2014; Seth, 2013; Seth et al., 2011; Seth and Tsakiris, 2018), although in the proposed view the role of interoception is reduced. Interoception typically operates on a longer temporal scale, and does not provide as clear pattern of statistical regularities differentiating between self- and externally-generated actions as motor behavior, making it a worse candidate for bootstrapping the primordial self-other distinction. However, it is likely that once the brain discovers this fundamental distinction through sensorimotor congruence, interoception becomes classified as an internal process, and as such becomes a constituting part of the representational self.

### 3.2 The auditory and first-person visual body representation

At the moment of birth, one’s stream of sensory stimulation changes. Once out of the womb, one begins to receive much more detailed visual input, while at the same time one’s tactile input drastically changes its nature (one is no longer submerged in uterine fluid). In regard to vision a foetus can only detect big differences in luminance (Eswaran et al., 2004; Peleg and Goldman, 1980), while a newborn becomes exposed to a wide variety of complex visual scenes. However, the scope of possible experiences increases in all sensory modalities, not only in vision.

This explosive increase of richness of experienced sensations means more data that the brain needs to make sense of. And it includes the possibility to classify elements of this sensory stream into self-related versus non-self-related. Importantly, a newborn needs to learn to distinguish between self- and other-generated sounds (e.g., crying), and between seen objects which are parts of the environment versus the ones which are parts of one’s body. I postulate that in both cases of vision and audition the underlying mechanism of learning to make the distinction between self and non-self will be similar to the one described in the previous section – the brain will need to pick up on the regularities between one’s motor activity and its corresponding auditory and visual consequences. The ones which reliably accompany one’s motor activity will then become represented as forming parts of one’s self. It may be the sound of one’s crying, or the image of one’s body parts moving as seen from the first-person perspective. Once learned, they become incorporated into the structure of internal representations of hidden causes which are classified as reflecting the self.

How quickly does a newborn learn that they can have agency over seen objects? Several studies suggest that this ability is developed during the first year (Kenward, 2010; Miyazaki et al., 2014; Zaadnoordijk et al., 2020), perhaps as early as in the second month of life (Rochat and Striano, 1999, 2000; Watanabe and Taga, 2006, 2009, 2011; Watson and Ramey, 1972). However, all of these studies investigated signs of agency over objects external to one’s body and utilized procedures in which interaction with these objects was very short (typically several minutes). It is possible that a newborn acquires agency over one’s seen body parts, especially hands, much earlier, as a consequence of the fact that they are much more reliably associated with one’s motor commands than any external object, and that newborns have much more time to learn it (virtually all of the waking life after birth). This possibility is further supported by findings showing that even newborns are sensitive to visuo-tactile congruency (even for images of faces: Filippetti et al., 2013; Filippetti et al., 2015), which is a necessary prerequisite for visual self-recognition.

**Figure 6:**
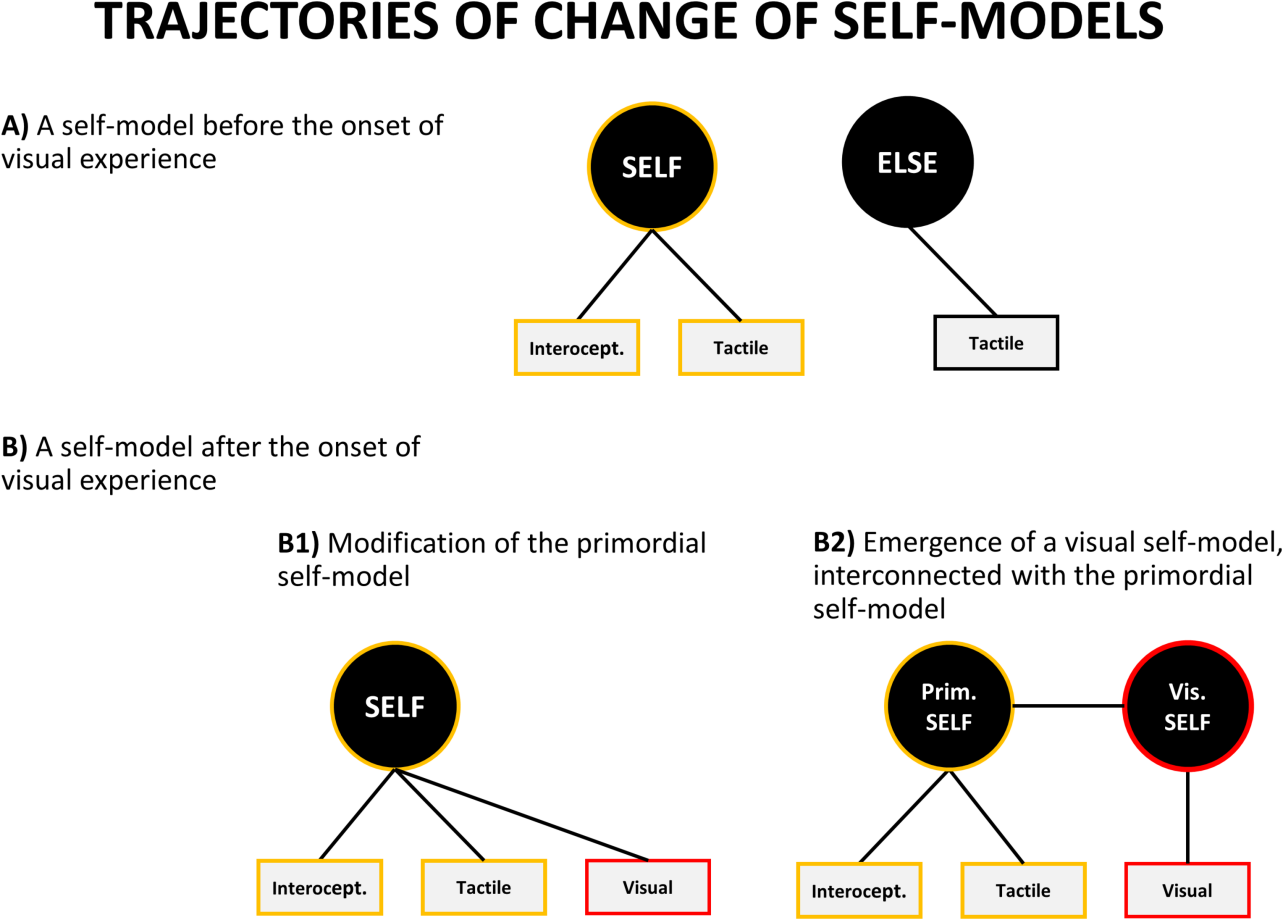
Theoretically possible trajectories of change of self-concept. However, as described in the text, empirical evidence favors a variant of model B2.

How do these new visual and auditory self-representations relate to the self-model which has been already developed in a foetus? There are several theoretical possibilities, but the two main ones are: either they get incorporated into the pre-existing self-model or new specialized self-models are being formed (Figure 6). In the first case, the pre-existing self-model becomes expanded to include new auditory and visual aspects. It means that no new model is formed – the old one is modified to accommodate additional modalities. Alternatively, new individual self-models might emerge for each modality or aspect of one’s self-representation.

What is important is that these two options lead to different empirical predictions. If each self-model is localized in a different part of the brain then people with lesions in different brain areas should selectively lose individual self-models. For example, it should be possible to find clinical conditions in which one selectively loses an ability to visually recognize one’s body from the first person perspective, while retaining the ability to use tactile and prioprioceptive information for self-identification. Although extremely rare, there are clinical cases providing preliminary indication that this situation might happen. Tobita et al. (1995) reported a case of a 52-year old man suffering from progressive cortico-basal degeneration (which began in the left parietal lobe, which is sometimes implicated as a crucial area for one’s body representation) who displayed the following symptoms:

He [the patient] could not point at any part of his own body in response to verbal or visual commands. On the other hand, he could point at every part of the examiner’s body or of the illustrated body image. Deep sensations and linguistic functions were not involved. This cognitive impairment was regarded as autotopagnosia. In contrast with inability to recognize any part of the own body in response to the commands, he could name every part of his body as soon as the examiner touched there. Moreover, his symptoms of autotopagnosia were ameliorated by looking at himself in a mirror; he could point at any part of his own body. Tobita et al. (1995, p. 296).

This case illustrates a condition in which the patient appears to have selectively impaired first-person visual body-representation, while had a preserved third-person visual self-representation (pointing was preserved when looking at one’s mirror reflection), and tactile representation (he could name his body parts after being touched).

A similar case, although limited to the left arm, was reported by Verret and Lapresle (1978). They described a patient, claiming that she did not have her left arm. At the same time, she was able to recognize it when touching it with her right hand or when seeing it in a mirror (but only when her left hand was not visible).

It is important to note that in both of the described cases the impairment manifested itself only in regard to the first-person visual representation of one’s body, and not for its third-person version – their mirror reflection. This suggests that these two visual representations of our bodies may be underpinned by separate internal self-models. Indeed, there are other reasons to believe that it is the case, including the ones which suggest that the third-person representation of one’s body comes as the next step of development of the self. On the other hand, the described case studies by themselves provide only preliminary indication of the full double dissociation that needs to be shown to provide convincing evidence for the proposed model. A full in-depth review of neurological data could provide such evidence – it is however beyond the scope of the current paper.

### 3.3 The third-person visual body representation

After the first year of life an infant knows at least two things about oneself: (1) that some things that one sees are parts of one’s body (forming the first-person visual self-representation), and (2) that some things that one sees are other living creatures, including other people. However, it seems that only until several months later an infant becomes capable to realize that in some specific situations these two things can come together in the form of one’s mirror image – something that looks like someone else but in fact is me.

From the point of view of an infant, when seeing one’s mirror image for the first time, one encounters a curious creature – something that looks just like any other infant, but is characterized by an uncommon attitude. This attitude manifests itself by the fact that this creature imitates our infant. Moreover, the creature is so skilled in this task that even though an infant tries as hard as possible, they can never surprise the creature by doing something that the latter cannot predict and perform as well. As human adults we know how to make sense of this situation – contrary to the appearance, what one sees in a mirror is not someone else, but oneself as seen from the third-person perspective. However, for an infant finding the solution to this conundrum seems to be much more difficult.

As illustrated by research conducted during the last 50 years, acquisition of the skill to recognize oneself in a mirror (MSR) is a process which takes place slowly and in stages (Amsterdam, 1972; Bertenthal and Fischer, 1978; Filippetti and Tsakiris, 2018; Lewis and Brooks-Gunn, 1979; Lewis and Ramsay, 2004; Schulman and Kaplowitz, 1977). At first (between 6 and 12 months), infants treat their reflection like a potential playmate (Amsterdam, 1972). However, they quickly become frustrated, possibly as a consequence of the fact that the reflection does not enter into meaningful interaction, and eventually begin to ignore the character in the mirror. It is only when they reach the age of between 18 and 24 months when they begin to exhibit clear signs of understanding of the nature of their mirror reflection, such as correctly naming it when asked by adults.

I want to propose that this developmental pattern can be explained as an instance of new model acquisition. At the outset, an infant possesses a visual first-person self-representation (one’s body as seen from the first-person perspective) and an internal model of another person (how another person behaves and looks from the third-person perspective). Once an infant encounters a mirror reflection (or any other reflection, e.g., on a water surface), she or he meets an agent which appears to be another person, but at the same time violates many expectations about how another person behaves. This leads to the build up of prediction error, which needs to be explained away. One can do it by simply avoiding mirrors or ignoring them (what corresponds to the active inference strategy: Friston, 2010; Friston et al., 2010), although this strategy will not solve the problem in the long run – one cannot avoid mirrors for the rest of one’s life. A better strategy is to either adjust the existing internal model of other people in order to account for such individuals, or to create a new model for this special class of individuals. However, in both of these cases the outcome is suboptimal – the peculiar behavior of the mirror creature remains unexplained. Only the emergence of a new model which combines the two models which are already in infant’s possession, i.e., of other people and of the self seen from the first-person perspective (“it’s how the others see me!”), provides the perfect fit to the sensory data. This is the internal model of oneself as seen from the third-person perspective. The task of an infant is to restructure one’s internal hypothesis space and form such model. Seen this way, mirror self-recognition becomes an intellectual struggle, requiring a strike of insight reflecting mental reorganization similar to out-of-the-box thinking required to solve other ill-posed problems (Knoblich et al., 1999; Lewis et al., 2018; Pezzulo et al., 2014; Sternberg and Davidson, 1995; Weisberg, 2015).

If mirror self-recognition is just an ill-posed problem then the ability to recognize oneself in a mirror should not rely on an innate cognitive module (Barrett and Kurzban, 2006; Samuels, 2012), but on more general cognitive skills. Therefore, it should be possible to teach it to many animals which are traditionally believed to be incapable of it. However, because these animals may possess weaker cognitive skills, it may require long-term extensive training in a heavily simplified setup. Traditionally it is assumed that among primates only great apes can exhibit mirror self-recognition (Suddendorf and Butler, 2013). However, recent studies found that with appropriately long training it is possible to teach this ability to rhesus monkeys (Chang et al., 2015; Chang et al., 2017). These results suggest that mirror self-recognition may rely on general learning mechanisms rather than cognitive modules acquired through evolution.

The view that mirror self-recognition is acquired through acquisition of a new internal model is also in line with conservative accounts of significance of acquisition of this skill. Rich views of MSR argue that it marks the beginning of self-awareness (Gallup, 1970; Gallup et al., 2014). Conservative views disagree with this and postulate that MSR is a much less profound cognitive skill and provide a number of arguments supporting it (Rochat and Zahavi, 2011; Suddendorf and Butler, 2013). A conservative view is also more compatible with data showing that mirror self-recognition marks only the beginning of the longer process of acquisition of one’s third-person visual representation. Toddlers which pass the classical rouge mark test with mirrors typically need several more months until they can do it with live video recordings, and it is only around the age of four when they are able to recognize themselves in a delayed video recording or on a photography (Povinelli et al., 1996; Suddendorf et al., 2007).

Is there evidence that the third-person visual representation of oneself is underpinned by distinct brain areas than other self-representations? Data described in the previous section suggest that it is possible to experience disruptions of the first-person visual representation without accompanied disturbances of the third-person self-representation (Tobita et al., 1995; Verret and Lapresle, 1978). Moreover, instances of mirror agnosia show that lesions of the parietal cortex can lead to selective loss of the ability to comprehend how a mirror works (Binkofski et al., 1999; Ramachandran et al., 1997). Are there cases of people who fail to recognize themselves in a mirror, even though they retain the ability to use mirrors otherwise? Indeed, there are rare reports of mirrored self-misidentification following brain lesions in the right hemisphere (Villarejo et al., 2011; also following hypnotic suggestion: Connors et al., 2012) suggesting that the third-person self-representation may be underpinned by different brain structures than the visual first-person self-model. However, as indicated in the previous section – more research is needed to fully evaluate this possibility.

### 3.4 Extended, social, and abstract self-representations

Self-representations described so far all reflect different aspects of the self understood as one’s body and are grounded in information coming from specific sensory modalities. However, our self extends beyond our bodily self and our senses. Non-bodily and abstract self-representations (group membership, nationality, religion, abstract beliefs about ourselves and our bodies) are standard targets of social and cognitive theories of self and identity, not only in cognitive science, but also in social psychology (Baumeister, 1999; Baumeister and Tice, 1986; Markus, 1977; McCall and Simmons, 1966; Tajfel, 1982), sociology (Goffman, 1956; Mead, 1934; Owens et al., 2010; Stryker, 1968, 2008), political psychology (Jenke and Huettel, 2016; Van Bavel and Pereira, 2018; Whitehouse, 2018) and political science (Hayward and Watson, 2010; Huddy, 2001; Kalin and Sambanis, 2018) to name a few. They can include such diverse entities as representations of one’s clothes and possessions, territory, family and friends, group membership, personality traits, episodic memories, and many others (James, 1890).

How can this type of self-representation emerge in development? It may appear that the underlying mechanism must be different from the one involved in development of the bodily self-representations which were described so far. However, I will argue that they can emerge as a consequence of applying the same general rule as postulated for the emergence of other self-representations, i.e., differentiating between self- and non-self-related information. In this case, however, the scope of possible self-relatedness extends beyond one’s physical body and beyond sensory information. As a consequence, the mechanism underlying differentiation cannot rely on sensorimotor congruence, because we do not have motoric control over abstract entities and control over extra-bodily physical entities is typically only temporary.

What then, can be the underlying mechanism? The answer advocated here is that humans likely possess innate forms of the extended and social self-models. These two self-models, together with the bodily self-models, may later form the basis for subsequent development of the abstract self-representation through similar processes of formation of new internal models to the one advocated in regard to the bodily selves. It can be summarized through three postulates:

1. Humans possess evolutionarily inbuilt precursors of the extended self-model
2. Humans possess evolutionarily inbuilt precursors of the social self-model
3. Abstract self-model emerges on the basis of the extended, social and bodily self-models

I will discuss each of these claims in the following three sections.

#### 3.4.1 The extended self

The extended self is usually defined as the aspect of the self composed of representations of one’s possessions (Belk, 1988, 2013; Kim and Johnson, 2014). At the most fundamental level, representing something as mine (possession over something) reflects a cognitive ability to differentiate between objects in the environment which belong to me and the ones which do not. As such, it reflects the same fundamental classification operation (into “me” and “not-me”) as the one described earlier, although in this case it classifies extracorporeal objects and entities.

Human toddlers begin to demonstrate understanding possession at a similar time to when they begin to show signs of mirror self-recognition, usually when they are between 18 and 24-month old (Fasig, 2000; Rochat, 2011; Rodgon and Rashman, 1976). At this stage they begin to use possessive pronouns (e.g., “Mine!”), and exhibit defensive behavior in regard to objects which belong to them. Given these indications, by roughly 2 years of age the majority of toddlers appear to develop the explicit concept of possession, and as a consequence, acquire a basic form of the extended self. The fact that it develops so late might suggest that an internal model of possession is an advanced, high-level ability. Moreover, because it appears only after a toddler begins to speak, it suggests that representing possession may require language and language-based reasoning.

If representing own possession is such an advanced cognitive achievement then it should be absent in non-human animals. Although it has been rarely directly discussed in biology, there are phenomena indicating that certain forms of this capacity might be in fact widespread in animal kingdom (Strassmann and Queller, 2014). One example of representing extra-corporeal entities as self-related is the phenomenon of territoriality. Territoriality reflects a situation in which an animal lives and hunts or forages on a certain territory, but also actively defends it when a different animal or group of animals (typically of the same species) enters it (Noble, 1939). In order to do it, an animal must be able to recognize one’s territory and to represent it as “mine,” and hence to possess at least some rudimentary “self-territory model.” Territoriality is widespread among not only vertebrates, but also invertebrates (Hinsch and Komdeur, 2017; Stamps, 1994) showing that a basic form of the extended self does not require neither language nor high-level cognition. Others examples of behaviors indicating presence of basic forms of extended self are nesting, food caching, and building structures serving to attract potential mates (Ancrenaz et al., 2004; Borgia, 1995; Kaplan, 2015; Plumptre and Reynolds, 1997; Strassmann and Queller, 2014). In all of these cases animals are able to differentiate between non-bodily objects that belong to them and the ones that belong to others, what constitutes the defining characteristic of the extended self.

Within the Bayesian framework the ability to perform discrimination in regard to a territory or a nest suggests that these animals possess internal models (representations) which allow them to classify their perceived environment as either “my” territory (or nest, food, etc.) or not. While such internal model are typically domain-specific and much more restricted than the full-blown human concept of possession, they show that evolutionary precursors of the extended self might be innate also in humans, and the fact that toddlers manifest it only during the second year of life is caused by late maturation of the underlying neural circuitry (see also: Nancekivell et al., 2018).

#### 3.4.2 The social self

Animal behaviors suggest that many of them also possess some forms of a primordial social self. The social self, in line with the definition adopted in this paper, can be understood as underpinned by internal models which allow to distinguish between creatures (typically belonging to the same species) which are in some way related to “me,” and the ones which are not. The existence of a form of the social self can be inferred if a human or an animal behaves in a distinctive way toward certain individuals (for example by defending them - typically one’s mate, offspring, or members of the same group), as contrasted to its behavior toward other individuals. This ability might be absent in many species. For example, many species of fish breed by spawning a colony of eggs which are abandoned after laying them. In such cases there is no need for mating individuals to be able to identify each other after the mating occurred (and in species with external fertilization even during the process). However, in many other species, especially mammals and birds, one or both parents take care of their offspring. In this case they exhibit a special set of behaviors toward their children, as opposed to any other young animals of the same species. It includes defending and feeding them, and in some cases even forms of teaching (Hoppitt et al., 2008; Thornton and Raihani, 2010). Some species which live in colonies developed complex mechanisms allowing parents to recognize children from sometimes thousands of pups, like in the case of Mexican free-tailed bats which use vocal and olfactory signals to recognize their pups (Balcombe, 1990; McCracken, 1984; McCracken and Gustin, 1991). Similar rule applies to one’s mates. Many species form long-lasting monogamous relationships in which animals cooperate in raising offspring for extended periods of time, sometimes for tens of years (e.g., parrots: Kaplan, 2015). In this case often a male provides food and defends only the female that takes care of his offspring. This behavior, however, requires the male to be able to identify his mate and represent her as such. It means that he must possess an internal model of the mate, as opposed to all other female individuals from that species.

In humans a form of the ability to distinguish between close others and strangers is present from birth. Newborn humans are able to distinguish their mother from other human females very early on, demonstrating a basic form of the social self (Bushnell, 2001; Field et al., 1984). At the age of around 6 months infants develop stranger anxiety (Brooker et al., 2013; Waters et al., 1975). They begin to react with signs of distress during encounters with unfamiliar people, even when mother or other caretaker is present. This behavior typically peaks between 6 and 12 months of age and then decreases in intensity. It suggests that at that age infants can distinguish close others and potentially dangerous strangers, a distinction which may foreshadow ingroup/outgroup classification (cf. Dunham et al., 2008), which forms the basis of group-based forms of the social self, such as different forms of group identity.

In summary, comparative evidence shows that some forms of the social self are widespread in the animal kingdom (as evidenced by selective parental care and mate defence). Moreover, developmental studies showing that human newborns can recognize their mothers suggest that a basic form of social self is present at birth in humans as well.^5^

#### 3.4.3 The abstract self

The abstract self includes representational content that goes beyond representations of one’s body and objects or agents in the extra-corporeal space. It includes a wide range of content, but generally it is composed of self-related representations encoded in semantic and episodic memory. This is the type of self which has been visualized by the majority of nodes in Figure 1. Although it is composed of more abstract content thea the notions of the self described so far, the criterion used to determine whether a given representational content forms part of the self or not remains the same: a given representation forms a part of one’s (abstract) self if it is represented as self-related.

As such, the abstract self forms a category which includes multiple notions of the self present in psychological, sociological, and related literature. It includes one’s content of autobiographic memory (Haslam et al., 2011; Klein et al., 2004; Nelson and Fivush, 2004; Wang, 2004), content of semantic autobiographical memory (Martinelli et al., 2013), representations of one’s personality traits – sometimes regarded as constituting the “psychological self” (Hu et al., 2016), and all other forms of semantic self-knowledge (Baumeister, 1999; Conway, 2005; Gillihan and Farah, 2005; Kihlstrom et al., 2003; Markus, 1977; Martinelli et al., 2013; Neisser, 1988). It can include linguistic representations of one’s emotional state, metacognitive judgments about oneself (including one’s cognition), and abstract judgments about one’s body. It also involves various types of identities: national, political, religious, gender, cultural - all of them are formed on the basis of socially and culturally transmitted information, and represent abstract socio-cultural constructs. The proposed model allows to link all of these seemingly disparate fields of study by showing that on the cognitive level they all reflect a manifold of representations stored in the semantic and episodic memory and acquired predominantly through mechanisms of social and cultural learning.

How such abstract self-representation can develop? The proposal is that it emerges based on the same mechanisms of new model acquisition as bodily self-models described before, i.e., by forming (and later reforming) an internal model which allows to classify abstract content into self and non-self. The crucial task for an individual is to learn that it can be applied to virtually any representational content, including highly abstract concepts and categories such as personality traits, philosophical ideas, etc. As argued in the previous sections, it is highly likely that humans are born with some basic capacity to represent objects in the environment (via possession - forming the basis of the extended self) and other people (via emotional attachment - underpinning the social self) as self-related. Moreover, very early in development human infants (but also many other animals) are able to distinguish between their body and the external world, at least when perceived from the first-person perspective. It appears safe to assume that before they begin to exhibit signs of linguistic abstract thoughts, young humans already possess a range of self-representations (bodily, social, and probably basic extended). The task for the developing toddler becomes to notice the underlying rule – that we can classify all representations as self versus non-self-related - and apply it to the emerging abstract representations as well.

The first clear indication that abstract self is emerging comes when one begins to use self-related language. It includes first instances of usage of personal and possessive pronouns (“I,” “Me,” “mine”) and being able to generate self-descriptions. In typically developing humans this usually happens between 18 and 24 month of life (Bates, 1990; Fasig, 2000; Levine, 1983; Stipek et al., 1990; Tomasello, 1998). This is the same period of time as when toddlers begin to recognize themselves in a mirror, but also when they begin to show explicit signs of understanding of the concept of possession (Fasig, 2000; Rochat, 2011). It raises the possibility that these three developmental achievements are related. However, a study by Fasig (2000), in which all three were tested found that while use of self-related language and understanding of possession tend to co-occur, mirror self-recognition was unrelated to them (see also: Levine, 1983). It suggests that in humans the emergence of the abstract self may be related to development of explicit understanding of possession. One possibility is that possession serves as a springboard for development of abstract self-representation. One way to investigate this hypothesis is to conduct longitudinal research tracking the developmental trajectory of personal and possessive pronouns individually, as well as self-descriptions (current methods usually group them together, e.g., Stipek et al., 1990) in order to determine which of them appear earlier and which later. If indeed possession is the basis for explicit linguistic self-representation then toddlers should first begin to understand and produce possessive pronouns (“my,” “mine”) and only later first-person personal pronouns.

Once the basic form of abstract self-representation emerges (as indicated by usage of self-related language) it becomes subject to further development as a result of socio-cultural learning and individual reasoning. This process can be compared to development of understanding of mathematics, which begins with understanding of individual numbers (one, two, three), then the concept of natural numbers, and then integers, rational and complex numbers (Carey, 2009). According to the proposed theory, abstract self-model changes during the course of human development in analogous way. It can be illustrated by the developmental trajectory of self-descriptions. Very young toddlers use only pronouns and own name for self description. However, as they mature their ability to describe themselves rapidly increases. Already before the fourth year of life one’s self-description can become very sophisticated, as illustrated by the following example:

I’m 3 years old, I’m a boy, and my name is Jason. I live with my mommy and daddy who really love me. My mommy makes me yummy spaghetti! I am going to get my own baby sister for Christmas! I have blue eyes and a kitty that is orange and a television in my own room, it’s all mine! I know all of my ABC’s, listen: A, B, C, D, E, F, G, H, J, L, K, O, P, Q, R, X, Y, Z. I can run real fast, faster than when I was 2. And I can kick a soccer ball real far, all the way from one end of the field to the other. I’m a lot bigger now. When I look in the mirror at me, I can tell I grew. My daddy puts marks on the mirror to show how much taller I get. I have a nice teacher at preschool, she thinks I’m great at everything! I can count up to 100, want to hear me? I can climb to the top of the jungle gym, I’m not scared! I’m never scared! I’m always happy. I’m really strong. I can lift this chair, watch me! My mommy and I like to make up stories about me, she helps me remember things I did or said. Harter (2012, p. 28).

While younger children use mostly concrete concepts referring to external observable characteristics to describe themselves (I’m a girl, I have blond hair, etc.), with time their self-descriptions begin to include more abstract and non-observable concepts (such as psychological traits: Yuill, 1992a, 1992b), and they become more structured and coherent (Damon and Hart, 1991; Harter, 2012). They also tend to take more narrative form, what marks the emergence of autobiographical memory (Klein et al., 2004; Nelson and Fivush, 2004), which forms the basis of what is often regarded as the narrative self (Dennett, 2014; Gallagher, 2000; Schechtman, 2011) or narrative identity (McAdams, 2001; McAdams and McLean, 2013).

The abstract self is a continuously developing collection of semantic knowledge and episodic memories. Importantly, this development is predominantly driven by socio-cultural learning mechanisms, as illustrated by the fact that self-descriptions differ across cultures from very young age (Hart and Edelstein, 1992; Wang, 2004, see also: Nelson and Fivush, 2004). However, the role of socio-cultural environment becomes even more apparent with age. For example, during adolescence, self-descriptions begin to increase in complexity and start to resemble adult ones by increasingly referring to specific sociocultural entities, such us cultural groups (subcultures, social classes), philosophical and social convictions (political and religious beliefs, personal beliefs), preferences (taste in music and movies) etc. As such, one’s abstract self becomes increasingly composed of representations of socio-cultural entities, such as nationality, religion, social position, or even spiritual beliefs, rather than the ones rooted in one’s body or the physical world. Finally, it may even include metacognitive representations, such as thoughts about one’s thought or one’s cognition – a situation which is especially vivid in mental disorders. For example in some types of schizophrenia an affected individual may develop beliefs that some of their thoughts belong to another person (Bortolotti and Broome, 2009; Martin and Pacherie, 2013; Young, 2008). In this case one begins to represent some of one’s thoughts as self-related, while the others as belonging to someone else.

A fully developed abstract self is specific to humans only, because according to the current knowledge no other animals possess language and means for cultural learning of highly abstract concepts. However, the question to what extent other animals can possess a basic form of the abstract self is difficult to address. It may be possible for an organism to acquire some form of an abstract self-model in the absence of language. If an animal can represent possession, or be able to recognize itself in a mirror, then perhaps it may be also capable to develop a basic form of the abstract self-representation.

## 4 Innate and learned models

The proposed model assumes that the brain performs approximate Bayesian inference. Under this approach the brain is seen as an inference machine which is composed of (hierarchically organized) internal models of hidden causes of sensory input (Clark, 2016; Hohwy, 2013). The proposed model suggests that there is a neural mechanism which allows new models to emerge as a result of recombination or modification of the existing models (see for example: Lake et al., 2018; Smith et al., 2019). As such, it postulates how new models can be acquired during the lifespan of an individual. However, cognitive representations can also be acquired in phylogeny, i.e., over the time course of multiple generations, as a result of evolution. Indeed, there is strong evidence that humans are born with an evolutionarily hard-wired stock of representations. For example, newborns appear to be able to detect faces (Johnson, 2005; Johnson et al., 1991), and to discriminate biological from non-biological motion (Bardi et al., 2011, 2014; Bottari et al., 2015). Moreover, a rudimentary preference for face-like stimuli can be detected in foetuses even before birth (Reid et al., 2017). These results strongly suggest that newborns possess basic forms of internal models of faces and biological motion. Because visual experience in the uterus is extremely limited, these representations could not have been acquired via mechanisms of learning. Instead, they must have been genetically encoded.

Genetic effects can be easily accommodated into Bayesian models of cognition in the form of innate models. For example, in predictive coding and related theory of free energy principle evolutionary effects are understood as instances in which models of hidden causes are obtained through evolutionary processes (Sims, 2017) and understood as “optimizing the agent’s model and priors through neurodevelopment and natural selection” (Friston, 2010). As such, the problem of innateness can be rephrased as the problem of which models are acquired through evolution, and which need to be learned.

**Figure 7:**
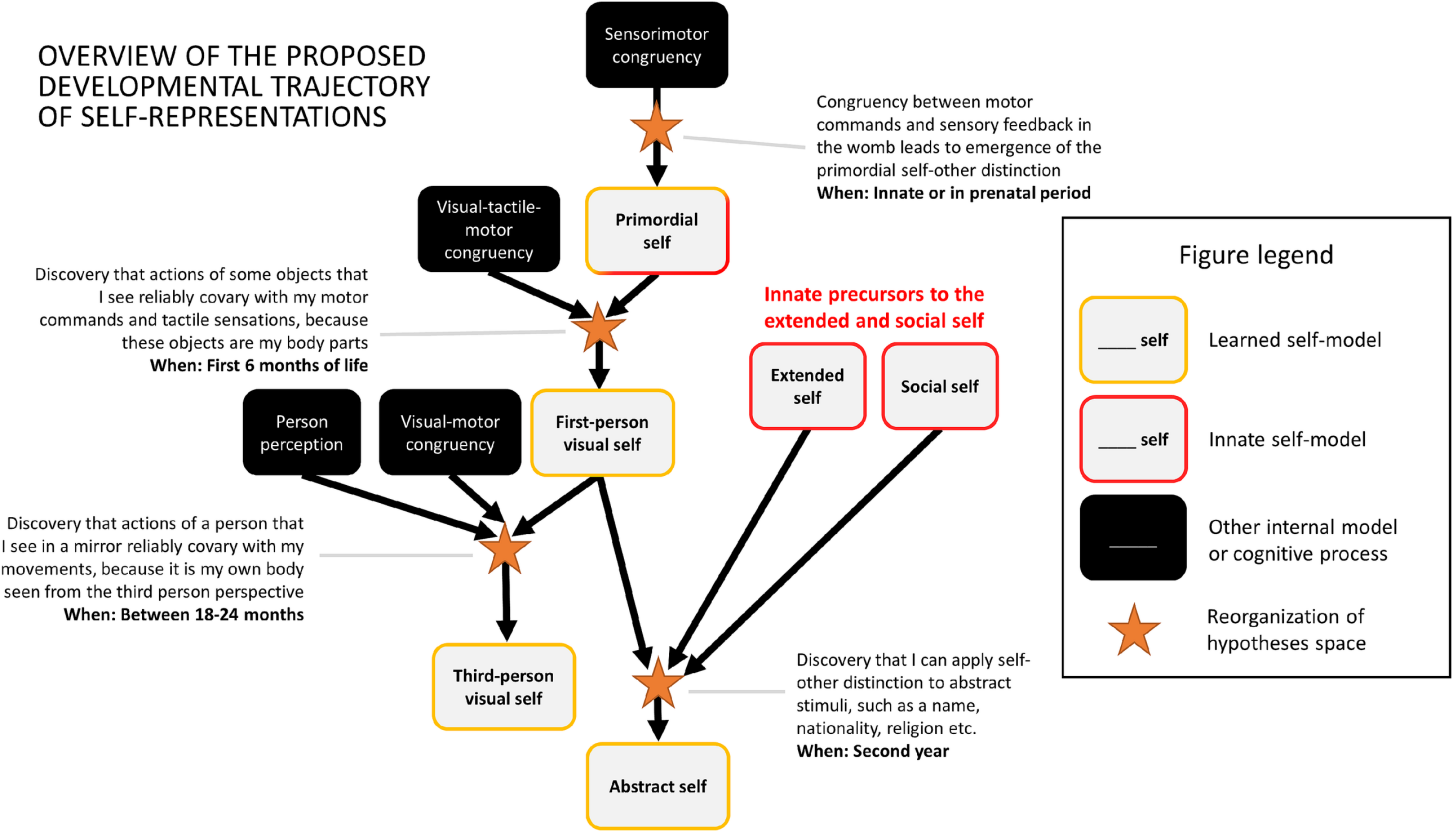
Overview of proposed stages of development of self-representations.

The proposal introduced in this paper aimed for maximal parsimony and therefore postulated innate models only where it seemed unlikely that learning plays a decisive role. These exceptions involve primitive forms of extended and social self which can be observed in multiple animal species, as manifested through, e.g., territorial behavior and defence of a mate or offspring (Figure 7). Moreover, these behaviors are manifested universally (i.e., often in almost all animals of a given species) and are highly stereotypical – which are characteristics of innately specified mechanisms (Gross and Rey, 2012). However, it is also possible that in humans they are learned – especially in the case of the extended self which becomes evident only around the second year of life.

It is also possible that some self-models which in principle could be learned, are inborn. Biological organisms can greatly benefit from having an innate stock of mechanisms and representations, including some types of internal models of the self. The proposed primordial self-other distinction is almost certainly innate in humans. One line of evidence comes from reports of phantom limb sensations in people with congenital absence of limbs, a condition which is also known as aplasic phantoms (Brugger et al., 2000; Gallagher et al., 1998; Melzack et al., 1997). Because such individuals do not possess limbs it was not possible for them to acquire parts of their sensori-motor self representing their missing limbs through learning. The fact that they can nevertheless experience phantom limbs suggests that these parts of self-representation must be genetically pre-programmed. However, there are alternative explanations of this phenomenon, which do not need one to postulate the existence of innate representations (Gallagher, 2006, chapter 4; Blumberg and Dooley, 2017). At present this debate is still unresolved.

At the same time, there are stages of development of the self which almost certainly are acquired through individual and cultural learning. There are strong reasons to believe that it includes mirror self-recognition and acquisition of abstract self-representation. In these cases mechanisms of new model acquisition proposed above could illustrate their full power. This might be especially true in regard to various high-level components of the abstract self-representation, such as national and religious identity, where various forms of cultural learning may be critical (Heyes, 2018; Mesoudi, 2011; Tomasello et al., 1993).

## 5 Empirical predictions and limitations

Bayesian approaches to cognition are sometimes criticized as being unfalsifiable (Bowers and Davis, 2012a, 2012b; Jones and Love, 2011), what raised a considerable discussion. Here I will follow the answer to it provided by Griffiths et al. (2012):

In evaluating claims about falsifiability, it is useful to distinguish between a model and a theoretical framework. A model is proposed to account for a specific phenomenon and makes specific assumptions in order to do so. A theoretical framework provides a general perspective and a set of tools for making models […] Models are falsifiable, but frameworks are typically not. Rather, frameworks live or die based on their ability to generate models that are useful. […] We believe that specific Bayesian models are readily falsifiable (or, at least, as falsifiable as any empirical hypothesis—any hypothesis can be “saved” by suitable *ad hoc* adjustments to other aspects of the theory […]). But the general Bayesian approach, as with any scientific framework, is not. Griffiths et al. (2012) p. 416.

The proposal of developmental trajectory of self-representations described in section 3 is a specific model that can be readily tested and the subsequent subsection will discuss how to do it. The general Bayesian framework is not falsifiable and it should be rather evaluated on the criteria of how useful for generating models it is. However, the current paper discusses not only a specific model of the developmental trajectory, but also a more general process of how development can take place in a Bayesian brain, which can be also treated as a smaller-scale framework. I will discuss it in the subsequent subsection.

### 5.1 How to test the model of development of self-representations

The proposal outlined in section 3 of this paper yields specific empirical predictions: if our self-representation is underpinned by multiple distinct internal models then we should observe evidence of double dissociations between these models. In principle, there should exist situations in which each individual postulated self-model is selectively present or absent. Phylogeny and ontogeny show examples in which more basic models are present, while more advanced models are absent, providing evidence for one part of this dissociation. An example is the dissociation between the first-person (1PVSR) and the third-person visual self-recognition (3PVSR). While 1PVSR is widespread in animal kingdom, the 3PVSR (as measured by mirror self-recognition) is not. Importantly, the same individuals (infants or animals) that at an earlier time point are not able to recognize themselves in a mirror, at a later point begin to display this ability. This illustrates that it is possible to possess either 1PVSR alone or to possess both. However, a complete proof of a double dissociation requires also evidence of a situation in which a developmentally earlier model is lost, while the later model is retained.^6^ This would rule out the possibility that the same model is responsible for both 1PVSR and 3PVSR, and that the emergence of mirror self-recognition only marks a developmental change in the internal model responsible for 1PVSR. While fully conclusive evidence is missing, there are neurological case studies (described in section 3.2 and 3.3: Fotopoulou et al., 2011; Tobita et al., 1995; Verret and Lapresle, 1978) that suggest that these two types of self-recognition are underpinned by two distinct models. However, more detailed analysis of the existing cases, as well as more research, are needed to validate this issue.

The same approach should be applied to each other pair of postulated models. The biggest challenge for the proposed theory lies in providing evidence of selective loss of earlier models, with preserved later models. This would mean, for example, demonstrating selective loss of aspects of the social or extended self, with otherwise intact abstract self-representation, or a selective loss of the primordial (sensorimotor) self-representation. Table 1 lists examples of evidence that would validate the proposed theory.

**Table 1:** Examples of actual and potential evidence for double dissociations between the postulated self-models.

Self-representation	Evidence of presence	Evidence (potential or existing) of absence, loss or disruption
Primordial (sensorimotor) self	Being able to distinguish between own body and the external world (objects, other agents) based on touch and proprioception	Clinical cases of loss of sense of body ownership: alien hand syndrome, anarchic hand syndrome, certain cases of somatoparaphrenia
First-person visual self	Successful recognition of one’s body parts when they are seen from the first-person perspective	Cases of selective disruption (Tobita et al., 1995; Verret and Lapresle, 1978). It is probably absent in congenitally blind individuals
Third-person visual self	Successful self-recognition in a mirror, an image or a video	Some cases of mirror agnosia. Developmentally absent in humans before 18 months. Absent in most non-human animals.
Extended self	Territorial behaviour, nesting, defence of one’s possessions, verbal reports of possession	It should be possible to observe loss of the ability to differentiate between self and other-owned objects, territory etc.
Social self	The capacity to preferentially treat one’s mate, offspring or group members.	It should be possible to observe loss of the ability to distinguish between close others and strangers (potentially in Capgras and Fregoli delusions)
Abstract self	The capacity to speak about abstract concepts and entities as being self-related	It should be possible to observe selective loss of the ability to use first-person personal pronouns. Abstract self is absent in animals and humans that do not possess the capacity for abstract thought or language.

### 5.2 Can we test the proposed developmental mechanism?

It is possible that the general logic of the proposed theory is correct, i.e., that self-representation emerges in a series of discrete steps through acquisition of new self-models, but that the specific trajectory from section 3 is wrong in respect to certain specific aspects. For example, it might turn out that the emergent models are not single, individual entities, but collections of several models. For example, it is possible that adult humans possess not one, but several distinct sensorimotor self-models, which might be composed of multiple models responsible for each half of the body or each body part. Similarly, humans might possess several third-person visual self-representations (one responsible for mirrors, another for video recordings, etc.). Further, it is similarly likely that there is not one model for abstract self-representation, but a collection of them, with each responsible for a different aspect of abstract self-representation. In a similar vein, there might be other independent self-models that do not fit neatly into the proposed classification, such as the agentive self proposed by Riva (2018).^7^ As such, the specific model proposed here will most likely require updating in the light of newly collected data. This, however, brings back the issue of falsifiability.

Understood in this way, the more general proposal introduced in this paper can be treated as a framework, rather than as a specific model. As argued by Griffiths et al. (2012) frameworks are generally not falsifiable. However, there are situations in which the proposed developmental mechanism can become validated. These would involve demonstrating that the basic assumptions of the framework are incorrect:

- The framework assumes that there is more than one self-model. If we observe that there is a single self-model that is responsible for all types of self-representations then this would falsify it.
- It assumes that at least some self-models emerge through learning. Demonstrating that all self-models (or self-representations) are innate would falsify the framework. Moreover, the framework is most useful if all self-models are learned. Conversely, the more self-models are shown to be innate the lower the explanatory power of the proposed framework.
- It assumes that we acquire new self-models through learning rather than maturation. Evidence that an organism possesses precursors to some self-model that gets activated by specific triggers would reduce the explanatory power of the framework. The most famous example of such mechanism is the phenomenon of imprinting (Hess, 1959; Lorenz, 1935), but similar mechanisms have been discussed for development of mirror self-recognition (Anderson and Gallup, 2015; Suddendorf and Butler, 2013).
- It assumes that we generally acquire new self-models through a specific form of learning, i.e., by recombining existing knowledge into new internal models.^8^ It means that evidence that we acquire new self-model only through different forms of learning could falsify the model.

Overall, the proposed framework describes acquisition of new self-models as a process that critically depends on a very specific form of learning, so any evidence of innateness, maturation, or alternative learning mechanisms directly undermine it.

## 6 Comparison with other theories

The proposed model of development of self-representation bears many similarities to the model recently proposed by Giuseppe Riva (2018). Both of these models postulate that the self emerges in a series of discrete developmental steps that lead to clearly distinguishable representations. Riva proposes six such representations: (1) the innate Sentient Body, which is an invariant spatial structure that integrates interoceptive, proprioceptive and vestibular signals and underpins the minimal phenomenal selfhood, (2) the Spatial Body that develops in the first 6 months of life and underpins self-location, (3) the Active Body that develops from the second half of the first year of life and underpins one’s sense of agency, (4) the Personal Body, forming one’s whole-body representation, which is responsible for the first-person reflective experience of owning a whole body (5) the Objectified Body, which stands for one’s third-person body self-representation and (6) the Social Body which integrates body-related social rules and narratives and underpins one’s sense of body satisfaction.

Many of these representations overlap with the current proposal: the primordial self is reminiscent of Riva’s Sentient Body and the postulated third-person visual self-representation corresponds to his Objectified Body. Moreover, Riva’s Social Body in the current proposal forms an important part of the abstract self-representation. However, in respect to the other developmental steps the two theories make different proposals. This comes as a consequence of differences in perspective: the current proposal differentiates self-representations (self-models) primarily based on types of information that underpin them: visual, auditory, tactile, abstract. On the other hand, Riva distinguishes body representations primarily based on the function that they play in constituting specific aspects of the self, i.e.: minimal phenomenal experience, self-location, agency, sense of whole body ownership, capacity for third-person self-reflection, and body satisfaction. This difference of perspective leads to important differences in how many and what types of self-representations one should postulate.

Other recent theories focused specifically on highlighting the role of social contact in development of the self. Humans spend the first 9 months of their development within the body of another person – their mother. It means that newly conceived humans are in direct social contact with another person from day one (even before they develop a neural system). This is a profoundly important insight and several recent papers put emphasis on it (Ciaunica, Constant, et al., 2021; Ciaunica, Safron, et al., 2021). While acknowledging its importance, the proposal outlined in this paper does not predict that this fact should make a fundamental difference to the emergence of the primordial self-model. In the postulated developmental mechanism outlined in section 3.1 the contrast that drives the emergence of the primordial self-model is between sensory stimulation that can be predicted by one’s motor commands versus stimulation that cannot be predicted. From this perspective the womb and the rest of mother’s body are likely treated as elements of the external world. However, it is possible that due to tight mechanical and physiological coupling with mother’s body parts of it can become represented by a foetus’ nervous system as part of oneself, and that this classification has important consequences in postnatal life.

Another important aspect raised in other recent theories is the role of sociality, and especially social touch, in development of self-representation. This topic has been recently extensively discussed in several papers (Atzil et al., 2018; Ciaunica and Fotopoulou, 2017; Fotopoulou and Tsakiris, 2017). In the current proposal social interaction is important in regard to the social, abstract and extended self: a person who is deprived of any social contact would likely fail to develop the social and extended self-models beyond what is genetically hard-wired. Moreover, the abstract self-model, which is fundamentally reliant on social and cultural learning, would be absent altogether. However, the current proposal does not treat social affiliative contact or social touch as necessary factors for development of any of the proposed self-models. It means that people who were in a social environment, but were deprived of physical contact with others (like children raised in orphanages in Romania under Nicolae Ceaușescu’s regime) should still be able to develop all of the postulated self-models, although deprivation of sociality and social touch, especially in childhood, might strongly affect how quickly and in what form each model develops. This might naturally have great impact on one’s social self, leading socially deprived people to develop altered self-representations (Carlson and Earls, 1997; Nelson III et al., 2007).

## 7 Conclusion

The current paper proposed a novel theory of the self understood as a representational structure in a Bayesian brain. By taking a developmental perspective it linked research traditions on bodily, abstract, social and extended self and proposed of a framework in which different facets of the self are understood as different stages of development of one’s internal self-models. The goal was, following the approach of William James (1890), to provide a comprehensive classification of different types of the self, but also to suggest how they may be related to each other. The theory advocated in this paper yields testable predictions regarding the structure of self-representation not only in humans but also in other animals. It allows to describe and explain selective losses of components of the self (e.g., first-person visual self-representation, third-person visual self-representation) and situate them within one general framework.

The proposed theory may be also regarded as a case study of how the process of acquisition of new internal models can be understood in the context of Bayesian models of cognition. It illustrates how using this framework may be beneficial. First, it provides a unifying account of diverse empirical phenomena, including (in the case of the self) bodily self-representation, social identity, territoriality, possession, and abstract self-related thoughts. Second, it suggests testable predictions which allow to test diverse models in order to determine the architecture underlying self-representations. In the interest of brevity this paper focused mainly on case studies of brain lesion showing dissociations between different types of self-models, as they provide the strongest evidence for specific selective impairments. However, other types of empirical evidence, such as neuroimaging and electrophysiology, can be equally important in further investigations of the structure of the self.

## Data Availability

The original contributions presented in the study are included in the article/supplementary material, further inquiries can be directed to the corresponding author.

## Footnotes

^1^For different recent conceptualisations of self-as-object see: Sui, J. and Gu, X. (2017). Self as Object: Emerging Trends in Self Research. *Trends Neurosci*, *40*(11), 643–653. https://doi.org/10.1016/j.tins.2017.09.002 and Truong, G. and Todd, R. M. (2017). SOAP Opera: Self as Object and Agent in Prioritizing Attention. *J Cogn Neurosci*, *29*(6), 937–952. https://doi.org/10.1162/jocn_a_01083

^2^In principle a categorical hypothesis space might contain almost an infinitely large number of categories. This might raise a concern that such mechanism might be computationally inefficient or even intractable. One solution to this problem has been provided by sampling-based approach to Bayesian cognition. Under this view we do not perform Bayesian inference over the full hypothesis space at once, but rather sample from it across time. It means that we might still entertain a very unlikely hypothesis if we spend enough time exploring the hypothesis space. This might happen if our sensory data is highly different than our prior distribution (what I see is very different from what I expected to observe) and I keep trying to find a satisfying explanation. For example, if I see a cat floating in the air in front of me I might try to explain it by going through otherwise unlikely explanations: am I dreaming? Am I in the Matrix? Was my food spiked with drugs? For sampling based approaches see: Fiser, J., Berkes, P., Orbán, G., and Lengyel, M. (2010). Statistically optimal perception and learning: from behavior to neural representations. *Trends in Cognitive Sciences*, *14*(3), 119–130. Orbán, G., Berkes, P., Fiser, J., and Lengyel, M. (2016). Neural variability and sampling-based probabilistic representations in the visual cortex. *Neuron*, *92*(2), 530–543.

^3^The origins of this idea can be traced back to the theory of assimilation and accommodation introduced by Jean Piaget Piaget, J. (2013). *The construction of reality in the child*. Routledge.

^4^It is likely that in practice only the explicit model of the self emerges, and the non-self model functions as an implicit model representing all the unexplained residual “noise.”

^5^Of course the basic human social self, as well as the basic extended self are most likely not direct evolutionary descendants of mechanism that led to the development of these basic social and extended selves in many other animals (especially the ones that are evolutionarily distant from us, like birds). It also means that specific mechanisms that underlie them (at the algorithmic and implementational levels) can be very different in humans.

^6^Because the developmentally later models are constructed based on the earlier models, for such dissociation to be observed the loss of an earlier model (for example due to a brain lesion) can occur only after the later-model has been already acquired.

^7^The model described in section 3 focused on self-representations that are relatively stable across time, as this is how this term is usually understood. However, it remains an open question whether it can be extended to describe how we represent our actions (what would underpin our sense of agency). In such case one would need to either postulate existence of an additional self-model or argue that it can be accounted by the already postulated self-models. More on sense of agency: Haggard, P. (2017). Sense of agency in the human brain. *Nature Reviews Neuroscience*, *18*(4), 196.

^8^An additional challenge is how to prove that new models can or have been acquired through creative recombination of the existing models. This issue is investigated within the area of study of cognitive processes underpinning creativity. However, it would require a more lengthy discussion that goes beyond the scope of this paper. See e.g.: Kaufman, J. C., & Sternberg, R. J. (2010). *The Cambridge handbook of creativity*. Cambridge University Press.
